# Supplementary material for: Intimate partner violence and unmet need for family planning in the Democratic Republic of the Congo: A secondary analysis of the moderating role of reproductive coercion using performance monitoring for action data
Source: PLoS One. 2026 May 5;21(5):e0331236. doi: 10.1371/journal.pone.0331236 (PMC13143080; doi:10.1371/journal.pone.0331236)
Supplement: S2 Table — (DOCX) [file pone.0331236.s002.docx]

| Model | χ² (df = 1) | p-value | Interpretation |
| --- | --- | --- | --- |
| Model 1a | 60.86 | 0.000 | Heteroskedasticity detected |
| Model 1b | 55.93 | 0.000 | Heteroskedasticity detected |
| Model 1c | 52.58 | 0.000 | Heteroskedasticity detected |
| Model 1d | 54.84 | 0.000 | Heteroskedasticity detected |
| Model 2a | 65.47 | 0.000 | Heteroskedasticity detected |
| Model 2b | 58.91 | 0.000 | Heteroskedasticity detected |
| Model 2c | 57.09 | 0.000 | Heteroskedasticity detected |
| Model 2d | 58.58 | 0.000 | Heteroskedasticity detected |

**Note:**
The Breusch–Pagan/Cook–Weisberg test evaluates the null hypothesis of homoskedasticity (constant variance of residuals). A p-value < 0.05 indicates rejection of the null hypothesis, suggesting the presence of heteroskedasticity. All tests were computed using the fitted values from each model.
