# Supplementary material for: Intimate partner violence and unmet need for family planning in the Democratic Republic of the Congo: A secondary analysis of the moderating role of reproductive coercion using performance monitoring for action data
Source: PLoS One. 2026 May 5;21(5):e0331236. doi: 10.1371/journal.pone.0331236 (PMC13143080; doi:10.1371/journal.pone.0331236)
Supplement: S3 Table — (DOCX) [file pone.0331236.s003.docx]

|  | **Model 1 ^a^** | | **Model 1 ^b^** | | **Model 1 ^c^** | |
| --- | --- | --- | --- | --- | --- | --- |
|  | **Coef.** | **95%CI** | **Coef.** | **95% CI** | **Coef.** | **95% CI** |
| **Experienced sexual violence** |  |  |  |  |  |  |
| No | Ref. |  |  |  |  |  |
| Yes | **0.12***** | **(0.04; 0.21)** |  |  |  |  |
| **Experienced physical violence** |  |  |  |  |  |  |
| No |  |  | Ref. |  |  |  |
| Yes |  |  | 0.07 | (-0.01; 0.15) |  |  |
| **Experienced emotional violence** |  |  |  |  |  |  |
| No |  |  |  |  | Ref. |  |
| Yes |  |  |  |  | -0.02 | (-0.07; 0.03) |
| **Couple’s level of education** |  |  |  |  |  |  |
| Lower - Lower | Ref. |  | Ref. |  | Ref. |  |
| High - High | -0.09 | (-0.21; 0.02) | -0.10 | (-0.22; 0.01) | -0.10 | (-0.21; 0.01) |
| Women better educated than men | -0.01 | (-0.20; 0.20) | -0.01 | (-0.21; 0.19) | 0.00 | (-0.20; 0.20) |
| Women with less education than men | -0.10 | (-0.23; 0.01) | -0.11 | (-0.23; 0.01) | -0.11 | (-0.23; 0.01) |
| **Age (group)** |  |  |  |  |  |  |
| 15 - 24 | Ref. |  | Ref. |  | Ref. |  |
| 25 - 34 | **-0.10**** | **(-0.16; 0.01)** | **-0.09**** | **(-0.16; -0.01)** | **-0.09**** | **(-0.17; -0.01)** |
| 35 - 49 | **-0.15***** | **(-0.23; -0.06)** | **-0.14***** | **(-0.23; -0.06)** | **-0.15***** | **(-0.24; -0.06)** |
| **Parity or number of children** |  |  |  |  |  |  |
| Nulliparous | Ref. |  | Ref. |  | Ref. |  |
| 1 - 2 children | 0.01 | (-0.10; 0.10) | 0.01 | (-0.09; 0.09) | 0.01 | (-0.08; 0.09) |
| 3 - 4 children | 0.09 | (-0.01; 0.18) | 0.09 | (-0.001; 0.18) | 0.09 | (0.002; 0.19) |
| 5 children + | 0.10 | (-0.01; 0.20) | **0.10**** | **(0.001; 0.21)** | 0.10 | (0.003; 0.21) |
| **Wealth tertile** |  |  |  |  |  |  |
| Lowest | Ref. |  | Ref. |  | Ref. |  |
| Middle | 0.01 | (-0.06; 0.07) | 0.01 | (-0.07; 0.07) | 0,01 | (-0.06; 0.07) |
| Highest | -0.03 | (-0.08; 0.03) | -0.03 | (-0.09; 0.02) | -0,03 | (-0.09; 0.02) |
| **Employment status** |  |  |  |  |  |  |
| Has not worked (last 12 months) | Ref. |  | Ref. |  | Ref. |  |
| Worked (last 12 months) | **-0.08***** | **(-0,13; -0.03)** | **-0.08***** | **(-0.13; -0.03)** | **-0.08***** | **(-0.13; -0.03)** |
| **Region of residence** |  |  |  |  |  |  |
| Kinshasa | Ref. |  | Ref. |  | Ref. |  |
| Kongo Central | 0.02 | (-0.04; 0.08) | 0,02 | (-0.04; 0.07) | 0.02 | (-0.04; 0.07) |
| **Religion** |  |  |  |  |  |  |
| No religion | Ref. |  | Ref. |  | Ref. |  |
| Christian | -0.01 | (-0.11; 0.12) | 0.01 | (-0.11; 0.12) | -0.01 | (-0.11; 0.12) |
| Other religions | 0.05 | (-0.08; 0.17) | 0.05 | (-0.07; 0.17) | 0.05 | (-0.07; 0.17) |
| Missing | -0.07 | (-0.28; 0.13) | -0.07 | (-0.27; 0.13) | -0.07 | (-0.27; 0.13) |

^a^ with experienced of sexual violence

^b^ with experienced of physical violence

^c^ with experienced of emotional violence

**^***^** p<0.01; **^**^** p<0.05; Ref.: reference; bolding indicates p < 0.05.
