# Supplementary material for: Intimate partner violence and unmet need for family planning in the Democratic Republic of the Congo: A secondary analysis of the moderating role of reproductive coercion using performance monitoring for action data
Source: PLoS One. 2026 May 5;21(5):e0331236. doi: 10.1371/journal.pone.0331236 (PMC13143080; doi:10.1371/journal.pone.0331236)
Supplement: Attachment — Submitted filename: Review (Plos One).docx [file pone.0331236.s004.docx]

Intimate Partner Violence, ‘Reproductive Sabotage’ and Unmet Need for Family Planning in the Democratic Republic of Congo: Evidence from Performance Monitoring for Action Data

The study addresses a highly important topic, and overall the manuscript is well written. However, some sections require minor revisions to enhance clarity, coherence, and overall presentation, making the article more compelling and polished.

**Abstract**

1. Short the abstract as per the journal’s guidelines (maximum limit of 300 words).
2. In the abstract, the abbreviation ‘DRC’ is used for the Democratic Republic of Congo, while in the conclusion, ‘RDC’ appears to refer to the same country. Please ensure consistency in abbreviation throughout the manuscript to avoid confusion.

**Introduction**

1. The title indicates that the study addresses both ‘reproductive sabotage’ and unmet need for family planning. However, the concept of ‘reproductive sabotage’ is not defined or discussed in the introduction or elsewhere in the manuscript. Please provide a clear explanation and contextual background of this term to align with the study’s focus.
2. In lines 105–106, a positive association between domestic violence and unmet need for family planning in sub-Saharan Africa (SSA) is reported without noting any exceptions. However, in lines 107–108, a negative association is mentioned. This contradiction should be clarified—please explain the context or differences in study findings to avoid confusion.

**Materials and Methods**

1. What is PMA in line 149? Please avoid using abbreviations without first defining them. Write out the full term followed by the abbreviation in parentheses upon first use, and then use the abbreviation consistently in subsequent sections.
2. Clearly explain how sample size was calculated.
3. Provide reference number of the ethical approval issued by the institute.

**Results**

Lines 302–303 include interpretation of the results, which should be reserved for the discussion section. Kindly relocate this interpretation to the discussion to maintain structural clarity.

**Discussion**

Interpret the results in the discussion section and elaborate further by integrating recent findings from other lower-middle-income countries to provide broader context and support for the study’s focus.

**Conclusion**

The conclusion should be concise, summarizing the key findings clearly and providing actionable recommendations based on the study results. Please consider shortening it to enhance focus and clarity.

Several grammatical and spelling errors were noted throughout the manuscript. These should be carefully reviewed and corrected to improve the overall readability and professionalism of the article
