# Supplementary material for: Intimate partner violence and unmet need for family planning in the Democratic Republic of the Congo: A secondary analysis of the moderating role of reproductive coercion using performance monitoring for action data
Source: PLoS One. 2026 May 5;21(5):e0331236. doi: 10.1371/journal.pone.0331236 (PMC13143080; doi:10.1371/journal.pone.0331236)
Supplement: Attachment — Submitted filename: Response to Reviewers.docx [file pone.0331236.s007.docx]

**Peer review report - Repartition of articles tasks for revision**

Journal Requirements:

When submitting your revision, we need you to address these additional requirements.

1. Please ensure that your manuscript meets PLOS ONE's style requirements, including those for file naming. The PLOS ONE style templates can be found at

[https://journals.plos.org/plosone/s/file?id=wjVg/PLOSOne_formatting_sample_main_body.pdf](https://journals.plos.org/plosone/s/file?id=wjVg/PLOSOne_formatting_sample_main_body.pdf) and [https://journals.plos.org/plosone/s/file?id=ba62/PLOSOne_formatting_sample_title_authors_affiliations.pdf](https://track.editorialmanager.com/CL0/https:%2F%2Fjournals.plos.org%2Fplosone%2Fs%2Ffile%3Fid=ba62%2FPLOSOne_formatting_sample_title_authors_affiliations.pdf/1/010f019984b753b0-6dfff73b-9de2-4af9-ade3-b54d49745222-000000/HJXfURezu-cgH4BpQqUxwM9Pv_cH0qn4jaHxFaKETFY=230)

2. We note that the grant information you provided in the ‘Funding Information’ and ‘Financial Disclosure’ sections do not match.

When you resubmit, please ensure that you provide the correct grant numbers for the awards you received for your study in the ‘Funding Information’ section.

3. Thank you for stating the following in your financial information:

[Université Catholique de Louvain  <http://dx.doi.org/10.13039/100007370> /Didier Mbombo Ndombe].

Please state what role the funders took in the study.  If the funders had no role, please state: "The funders had no role in study design, data collection and analysis, decision to publish, or preparation of the manuscript."

If this statement is not correct you must amend it as needed.

Please include this amended Role of Funder statement in your cover letter; we will change the online submission form on your behalf.

If the reviewer comments include a recommendation to cite specific previously published works, please review and evaluate these publications to determine whether they are relevant and should be cited. There is no requirement to cite these works unless the editor has indicated otherwise.

Response: The funders had no role in study design, data collection and analysis, decision to publish, or preparation of the manuscript.

**5. Review Comments to the Author**

Please use the space provided to explain your answers to the questions above. You may also include additional comments for the author, including concerns about dual publication, research ethics, or publication ethics. (Please upload your review as an attachment if it exceeds 20,000 characters)

***************************Reviewer 1****************************

**Reviewer #1**: I personally acknowledge your efforts. I have attached the comprehensive report plz take your time and revise few things for the next, plz correct the language and the spellings according to standard of PLOS one. I hope you may understand the query and will submit again well in time
regards

The study addresses a highly important topic, and overall the manuscript is well written. However, some sections require minor revisions to enhance clarity, coherence, and overall presentation, making the article more compelling and polished.

**Abstract**

1. Short the abstract as per the journal’s guidelines (maximum limit of 300 words).

Response: Thank you, we have shortened the abstract to 300 words.

2. In the abstract, the abbreviation ‘DRC’ is used for the Democratic Republic of Congo, while in the conclusion, ‘RDC’ appears to refer to the same country. Please ensure consistency in abbreviation throughout the manuscript to avoid confusion.

Response: Thank you for this remark. The correct abbreviation is DRC and is now consistently used.

**Introduction**

1. The title indicates that the study addresses both ‘reproductive sabotage’ and unmet need for family planning. However, the concept of ‘reproductive sabotage’ is not defined or discussed in the introduction or elsewhere in the manuscript. Please provide a clear explanation and contextual background of this term to align with the study’s focus.

Response: Thank you for this remark. The expression ‘reproductive sabotage’ has been omitted from the title. The present title is: “**Intimate Partner Violence and Unmet Need for Family Planning in the Democratic Republic of Congo: The Moderating Role of Reproductive Coercion**”.

2. In lines 105–106, a positive association between domestic violence and unmet need for family planning in sub-Saharan Africa (SSA) is reported without noting any exceptions. However, in lines 107–108, a negative association is mentioned. This contradiction should be clarified—please explain the context or differences in study findings to avoid confusion.

Response: We have added clarifications to the text. The first study (lines 105–106) used pooled data from 26 SSA countries, whereas the second study relied on pooled data from only six SSA countries (Cameroon, Kenya, Malawi, Rwanda, Uganda, and Zimbabwe).

**Materials and Methods**

1. What is PMA in line 149? Please avoid using abbreviations without first defining them. Write out the full term followed by the abbreviation in parentheses upon first use, and then use the abbreviation consistently in subsequent sections.

Response: The PMA mentioned in line 149 refers to Performance Monitoring for Action. It is already defined in the abstract, on line 25. We have redefined it in the data source section.

2. Clearly explain how sample size was calculated.

Response: We have added a sampling flow diagram as supplementary material and included the following text to explain how the sample size was calculated: “The sampling flow diagram is provided in S1 Fig.” We have also added further details regarding the sample design used in the PMA project.

We have added the following text: “The sample was designed to provide province level estimates of modern contraceptive prevalence rate among all women, with a margin-of-error of 5% at the province level. In each cluster, 35 households were randomly selected for interview, and all women aged 15–49 years in the selected households were eligible for the survey”.

3. Provide reference number of the ethical approval issued by the institute.

Response: The PMA project received ethical approval from the Institutional Review Boards (IRBs) at the Johns Hopkins Bloomberg School of Public Health (#14590) in November 2021 and the Kinshasa School of Public Health (KSPH) (#ESP/CE/159B/2021) in October 2021.

We have provided this information in the ethical approval section.

**Results.** Lines 302–303 include interpretation of the results, which should be reserved for the discussion section. Kindly relocate this interpretation to the discussion to maintain structural clarity.

Response: We have relocated this text to the discussion.

**Discussion.** Interpret the results in the discussion section and elaborate further by integrating recent findings from other lower-middle-income countries to provide broader context and support for the study’s focus.

Response: Thank you. We have relocated to the Discussion section the portions of the Results that were previously discussed at the end of the Results section. We have also added recent findings from lower-middle-income countries and/or sub-Saharan Africa that relate to our study, in order to provide broader context.

**Conclusion.** The conclusion should be concise, summarizing the key findings clearly and providing actionable recommendations based on the study results. Please consider shortening it to enhance focus and clarity. Several grammatical and spelling errors were noted throughout the manuscript. These should be carefully reviewed and corrected to improve the overall readability and professionalism of the article.

Response: Thank you for this remark. We have shortened the conclusion and corrected the grammatical and spelling errors noted throughout the manuscript.

***************************Reviewer 2****************************

**Reviewer #2**: This is a well-structured study that examines the link between intimate partner violence and unmet need for family planning in the DRC, uniquely highlighting reproductive coercion as a key modifier, using PMA data, sound methodology and offers critical public health insights for integrated IPV and reproductive health interventions. Below are a few recommendations for improvement:

1. **Statistical Transparency**: While the use of linear probability models (LPM) is appropriately, the manuscript would benefit from explaining why interaction effects were only assessed for reproductive coercion and not for other potential effect modifiers such as education or wealth. This addition could help rule out alternative moderating factors. Furthermore, while the results of the Breusch-Pagan/Cook-Weisberg test for heteroskedasticity not presented. Reporting these findings either within the main text or as supplementary material would enhance methodological transparency.

Response: Thank you for the comment. We did not assess interactions for other potential effect modifiers because this was not the objective of the analysis. The analysis was designed to test the interaction only for reproductive coercion. Moreover, we tested the interactions with these two variables out of curiosity; however, the results were not statistically significant.

We have added the results of the Breusch-Pagan/Cook-Weisberg test for heteroskedasticity as supplementary material.

2. **Causal Interpretation**: The authors appropriately note the limitation of the study's cross-sectional design in precluding causal inference. While this is a typical limitation, the discussion would be enhanced by suggesting concrete approaches for future research such as longitudinal study designs or leveraging follow-up waves of PMA data to better examine causal relationships over time.

Response: We believe that we have already addressed this aspect in lines 476–478, where we suggest that future research should use a longitudinal study design to more appropriately address causal inference.

3. The manuscript briefly acknowledges the possibility that a woman's intention to limit fertility may itself provoke IPV; however, this alternative hypothesis or causal pathway is insufficiently examined. Expanding the discussion to more fully consider the potential **bidirectional nature of the relationship** between IPV and fertility preferences would add valuable nuance and depth to the analysis.

Response: We have clarified and provided references regarding the potential bidirectional nature of the relationship between IPV and fertility preferences.

Qualitative research conducted in Kenya [44] supports this alternative assumption. The results showed that partners used IPV and/or reproductive coercion in response to women’s use of contraception to limit births. Some partners accused women of engaging in prostitution or having extramarital affairs as the reason for taking contraceptive pills.

4. **Generalizability**: While the study clearly notes its focus on Kinshasa and Kongo Central, the discussion would benefit from elaborating on how regional socio-cultural, economic, or health system differences might limit broader applicability across the DRC or SSA.

Response: We have provided some explanations for why the results do not allow for national generalization. For example, sociocultural specificities vary greatly across provinces, and some are characterized by limited accessibility to health services.

5. **Under-reporting of IPV**: Strengthen the discussion by outlining PMA’s measures to reduce under-reporting, such as interviewer training, use of private settings, and assurance of confidentiality, to enhance confidence in the reliability of self-reported IPV data.

Response: We have provided the measures that PMA implemented to build trust with participants during interviews and data collection.

6. **Editorial Corrections**: Ensure consistent use of terminology (e.g., “DRC” instead of “RDC”), correct minor typographical errors, and verify that all citations accurately reflect the studies they reference to maintain clarity and scholarly accuracy.

Response: We have corrected the grammatical and spelling errors noted throughout the manuscript.

7. **Contextualization of Findings**: The authors should further explore why physical and emotional IPV were not associated with unmet need for family planning, in contrast to findings from other regions. Discussing possible cultural norms, reporting biases, differences in measurement, or the unique social dynamics of the DRC could offer important context and help explain these discrepancies for readers.

Response: We have added this text to clarify this contrast: “Varied sampling strategies and contextual factors may help explain these divergent findings. For instance, in the DRC, more than half of women consider wife-beating acceptable under certain circumstances. However, this attitude toward the acceptability of violence is not homogeneous, and substantial contextual and cultural differences exist both within and across countries”.

***************************Reviewer 3****************************

**Reviewer #3**: The central focus of this manuscript is to examine the association between domestic violence (binary, or multi-category variable) and unmet needs for family planning (a well-defined binary variable/response), in a sample of study subjects collected within the Democratic Republic of Congo. The study objectives are on target. My comments, primarily statistical, are as follows:

(a) It's not clear to me why the authors chose to consider **linear probability models** (LPM) for their analysis, as alternatives to the well-explored logistic regression approach, now adjusted for survey weighting. The LPMs, in my opinion, are never appealing for modeling discrete (here, binary) response, given that they may encounter range issues, i.e., the predicted probability can be > 1, or < 0. A lot of software (such as, the R survey package) can easily conduct logistic regression, with survey weights, and the basis of going the LPM route is not clear.

Response: We justified the choice of this model by adding the reference to Mood (2010), which compares estimates from several models, including logistic models and linear probability models (LPMs). This article highlights that logistic regression estimates do not behave in the same way as linear regression estimates, as they are affected by omitted variables—even when those variables are not related to the independent variables included in the model. *First*, it has been demonstrated that the interpretation of odds ratios can be biased due to unobserved heterogeneity, which includes control variables that are unmeasured, measurable but not included, or simply omitted from the model. *Second*, comparing odds ratios across models that contain different independent variables is problematic, because unobserved heterogeneity may vary from one model to another. *Third*, comparing odds ratios across samples, groups within samples, or over time is also problematic, even when the models use the same independent variables, since unobserved heterogeneity may differ across samples, groups, or time periods.

We acknowledge that one of the limitations of the LPM is that predicted probabilities may exceed 1 or fall below 0, which is theoretically impossible. However, Mood (2010) emphasizes that this is not usually a major concern, especially if compared to disadvantages mentioned above.

The LPM was therefore preferred because of its ability to address issues encountered with the logistic model, particularly the problem of unobserved heterogeneity when comparing several models [55].

[55] Mood C. Logistic Regression: Why We Cannot Do What We Think We Can Do, and What We Can Do About It. European Sociological Review. 2010;26: 67–82. doi:10.1093/esr/jcp006.

(b) While constructing the primary independent variable ("domestic violence"), the three main variables (sexual, physical and emotional) were considered as dummy (1/0), and thereafter, a global variable was constructed after summing up the 3 binary components. It's not clear if this "**global variable**" was used alongside the 3 dummy constructs in the same regression model. Such a route can induce inherent confounding.

Response: We confirm that we created a global variable (“any IPV”) by summing the three IPV components. However, this variable was not included in any model together with one of its individual components, since that component is already part of the global variable. In lines 344–347, we specified that Table 3 presents the results for each of these components, with each row corresponding to an independent model. Moreover, none of these variables appears in the list of covariates at the bottom of the table.

Additionally, to avoid any confusion, we have revised Table 3 by adding rows to clearly indicate that it includes four different models, and we have assigned a reference to each of them (Models 1a, 1b, 1c, and 1d).

(c) The discussion section should mention that the current findings are only based on the sample derived from the **DRC region**, and should allude to future studies with larger sample sizes across various regions to study the generalizability of the association measure.

Response: Thank you for the suggestion. We have incorporated this recommendation into the revised version.
