# Supplementary material for: Intimate partner violence and unmet need for family planning in the Democratic Republic of the Congo: A secondary analysis of the moderating role of reproductive coercion using performance monitoring for action data
Source: PLoS One. 2026 May 5;21(5):e0331236. doi: 10.1371/journal.pone.0331236 (PMC13143080; doi:10.1371/journal.pone.0331236)
Supplement: Attachment — Submitted filename: REMARKS PLOS ONE.docx [file pone.0331236.s006.docx]

**REMARKS**

Despite the revisions made to address the reviewers’ remarks, several areas still require further improvement. The following specific suggestions are recommended to enhance the clarity, accuracy, and overall quality of the manuscript:

- **Language and Grammar**: This manuscript requires improvement in English language usage and grammatical accuracy through thorough proofreading.
- **Abbreviation Usage:** The manuscript should ensure that each abbreviation is defined at its first occurrence in the text, after which the abbreviation may be used consistently in subsequent sections. Line 104 &117 check for IPV.
- **Novelty Claim & Clarity of Contribution:** The manuscript’s claim of being the first study on domestic violence and unmet need for family planning in the DRC is overstated. The authors cite prior research from Congo addressing similar issues, which undermines the assertion of novelty. The paper should clarify how it differs from existing studies and highlights its unique contribution without overstating originality.
- **Methodology Link:** The provided link for full details of the methodology (<https://fr.pmadata.org/data/survey-methodology>) is not accessible and should be corrected or replaced with a functional source.
- **Sample Size Consistency:** Information about the sample size is inconsistent with the details presented in S1 Fig.
- **Study Variables:** How authors differentiated between sexual violence and reproductive coercion? How reproductive coercion was assessed
- **Study Setting and Population:** More detailed information should be incorporated into the Methods section to provide better context and understanding.
- **Data Collection Process:** The data collection process remains unclear. While the ethical approval section suggests primary data collection with training on confidentiality and informed consent, the Materials and Methods section describe the use of secondary data from the PMA database, creating inconsistency that should be clarified.
- **Statistical Analyses:** Line 273 and 260 inconsistencies between thresholds of p value.
- **Results:**

1. **Line 304:** The reported percentage of domestic violence is 32.5%. Please clarify how this figure was calculated and whether it is based on weighted or raw data.
2. **Lines 306–312:** It is unclear whether these belong to the *Results* section or the *Discussion*. Kindly specify and ensure consistent section placement.
3. **Table 1 (Line 313):** The percentages for experienced reproductive coercion are reported as 11.9% and 11.4%, which appear inconsistent. Please verify and correct.
4. **Lines 332–333:** Table 1 does not contain information on the prevalence of unmet need for family planning, yet these lines reference such data. Please reconcile this discrepancy.
5. **Lines 332–351:** A table summarizing these results is missing. Consider adding the appropriate table for clarity and completeness.
6. **Table 2:** The total N=294, but the sum of categories equals 298. This suggests a possible typographical or data-entry error. Please review and correct.
7. **Line 356**: Reference is made to “S3 Table,” but this table is not provided. Kindly include or clarify.

- **Abstract:** Line 30 define past years. How authors concluded this because last 12 months was used in the data analyses. Conclusion needs improvements.
- **General:** The manuscript uses the terms domestic violence and sexual violence interchangeably, which creates conceptual confusion. Since the findings specifically pertain to sexual violence, the terminology should be standardized throughout the text to avoid misinterpretation. Furthermore, the title should be revised to explicitly reflect that the study focuses on sexual violence, ensuring consistency between the reported results and the manuscript’s framing.

Implementing these improvements will help ensure the manuscript is more polished, precise, and aligned with scholarly standards.
