# Supplementary material for: Intimate partner violence and unmet need for family planning in the Democratic Republic of the Congo: A secondary analysis of the moderating role of reproductive coercion using performance monitoring for action data
Source: PLoS One. 2026 May 5;21(5):e0331236. doi: 10.1371/journal.pone.0331236 (PMC13143080; doi:10.1371/journal.pone.0331236)
Supplement: Attachment — Submitted filename: Responses to reviewers2.docx [file pone.0331236.s008.docx]

**RESPONSE TO THE ACADEMIC EDITOR:**

1. The quality of **written english** is of concern and the author should pay attention to this. This should be done after the manuscript is revised before submission.

**Response:** We ensured that the English was edited by a professional service.

2. The **title** of the manuscript should be rewritten to include the study design

**Response:** The title was revised to explicitly reflect the study design, although we prefer to avoid using the expression “secondary analysis” here to simplify the title.

3. Under the **abstract**, the **study design** should be clear.

**Response:** The abstract was revised to explicitly reflect the study design, i.e., a secondary analysis of PMA data.

4.A. Under the **discussion section,** the strenght of the study should come before the limitation

**Response:** Done.

4.B. While **unanswered questions or future studie**s should be placed before conclusion not under limitation of study as it is.

**Response:** Done.

4. C Also, there should be a **section on implication of findings** to healthcare professionals and policy makers. This is different from recommendation.

**Response:** We reorganized and rewrote the final sections to respond to this comment.

5. The **conclusion** should be based on the findings from this research and the stated objectives. Avoid overgeneralization and make it concise.

**Response:** Done.

6. the **references** are not consistent, pay attention to this especially the use of uppercase letters. **Response:** Done. The references are managed automatically by Zotero, and the issue arises from the journal’s style specifications, which require the use of uppercase letters. We attempted to modify this formatting manually.

**REMARKS**

Despite the revisions made to address the reviewers’ remarks, several areas still require further improvement. The following specific suggestions are recommended to enhance the clarity, accuracy, and overall quality of the manuscript:

- **Language and Grammar**: This manuscript requires improvement in English language usage and grammatical accuracy through thorough proofreading.

**Response:** We ensured that the English was edited by a professional service.

- **Abbreviation Usage:** The manuscript should ensure that each abbreviation is defined at its first occurrence in the text, after which the abbreviation may be used consistently in subsequent sections. Line 104 &117 check for IPV.

**Response:**

The article has been submitted for English editing before resubmission. We have also systematically reviewed and rectified all issues related to the use of abbreviations. With respect to violence, we chose to use the term “intimate partner violence” exclusively instead of “domestic violence” and have applied this expression consistently throughout the document.

- **Novelty Claim & Clarity of Contribution:** The manuscript’s claim of being the first study on domestic violence and unmet need for family planning in the DRC is overstated. The authors cite prior research from Congo addressing similar issues, which undermines the assertion of novelty. The paper should clarify how it differs from existing studies and highlights its unique contribution without overstating originality.

**Response:**

Thank you for this remark. We confirm that our study is the first in the DRC. However, we have rephrased the sentence in the introduction as follows: “To the best of our knowledge, this study is the first to examine the relationship between IPV and unmet need for family planning specifically in the DRC, as previous studies on SSA considered pooled data [18] or countries other than the DRC [22].”

- **Methodology Link:** The provided link for full details of the methodology (<https://fr.pmadata.org/data/survey-methodology>) is not accessible and should be corrected or replaced with a functional source.

**Response:**

We have incorporated the appropriate bibliographic citations and removed the original hyperlink.

- **Sample Size Consistency:** Information about the sample size is inconsistent with the details presented in S1 Fig.

**Response:**

Thank you for this remark. The previous figure contained an error: the correct number is 1,387. We have revised Fig. S1 to include an additional stage that clarifies how 1,382 observations were retained for the final analysis. In this step, we excluded 5 observations that lacked complete information on the variables included in our models.

- **Study Variables:** How authors differentiated between sexual violence and reproductive coercion? How reproductive coercion was assessed.

**Response:**

Both reproductive coercion and sexual violence are defined in the methodology section. Reproductive coercion refers to the exertion of control over women’s reproductive autonomy through coercive behaviors, including but not limited to sabotaging contraceptive use and pressuring women to become pregnant. Sexual violence is conceptualized as one dimension of intimate partner violence and encompasses both nonphysical and physical forms of coercion, including pressuring or insisting on sexual intercourse when it is not desired (without the use of physical force) and physically forcing an individual to engage in sexual intercourse.

- **Study Setting and Population:** More detailed information should be incorporated into the Methods section to provide better context and understanding.

**Response:**

We have included slightly detailed information in the Methods section. We modified the paragraph as follows: “For the purposes of this study, the analytical sample included women in unions residing in the DRC, specifically in the provinces of Kinshasa and Kongo Central, where the PMA project was being implemented. Kinshasa is a densely populated, predominantly urban capital with relatively better access to health services and information, while Kongo Central is distinguished by a substantial predominance of rural areas and faces significant inequalities in access to essential resources and services. With the objective of examining the association between intimate partner violence (IPV) and unmet need for family planning, the study population was restricted to women aged 15–49 years living in a union, that is, women who are at risk of pregnancy or presenting reproductive potential. In addition, the analyses were limited to women in unions who responded to the module on violence against women and girls to capture information on IPV. All other individuals were excluded from the analytical sample using the “marital status” variable.”

- **Data Collection Process:** The data collection process remains unclear. While the ethical approval section suggests primary data collection with training on confidentiality and informed consent, the Materials and Methods section describe the use of secondary data from the PMA database, creating inconsistency that should be clarified.

**Response:**

Thank you for this remark. In the abstract and “data source” section, we now stress that we are using **secondary data** from the PMA. We also removed the description of the ethical approval process. This section was requested by one of the reviewers, but it can create inconsistency. In the “data source” section, we now more synthetically state that the PMA research received ethical approval, and we added a reference for it.

- **Statistical Analyses:** Line 273 and 260 inconsistencies between thresholds of p value.

**Response:**

The level of statistical significance was set at p < 0.05. We have made the necessary	corrections. Thank you.

- **Results:**

1. **Line 304:** The reported percentage of domestic violence is 32.5%. Please clarify how this figure was calculated and whether it is based on weighted or raw data.

**Response:**

We have clarified how the calculation was performed and have refined the column descriptions by explicitly specifying the denominators.

2. **Lines 306–312:** It is unclear whether these belong to the *Results* section or the *Discussion*. Kindly specify and ensure consistent section placement.

**Response:**

Thank you for noticing this redundancy. We maintained these results only in the *Results* section.

3. **Table 1 (Line 313):** The percentages for experienced reproductive coercion are reported as 11.9% and 11.4%, which appear inconsistent. Please verify and correct.

**Response:**

Thank you. We have systematically reviewed this section and corrected all the identified issues. The correct value is 11.4%.

4. **Lines 332–333:** Table 1 does not contain information on the prevalence of unmet need for family planning, yet these lines reference such data. Please reconcile this discrepancy.

**Response:**

Table 1 presents information on the prevalence of unmet need for family planning according to each category of the dependent variables. We added a note in Table 1 to clarify this point. The prevalence of unmet need is presented in Table 2. The tables are correctly cited in the text.

5. **Lines 332–351:** A table summarizing these results is missing. Consider adding the appropriate table for clarity and completeness.

**Response:**

Table 1 provides a comprehensive summary of these results. Columns N and % indicate the distribution of each covariate. The last column of Table 1 indicates the prevalence of unmet need for family planning according to each category of the dependent variable.

6. **Table 2:** The total N=294, but the sum of categories equals 298. This suggests a possible typographical or data-entry error. Please review and correct.

**Response:**

Thank you for noticing this error. We have systematically reviewed this section and corrected all the identified issues. The correct value is 298. We corrected Table 2 accordingly.

7. **Line 356**: Reference is made to “S3 Table,” but this table is not provided. Kindly include or clarify.

**Response:**

Table S3 is now provided as supplementary material; it contains comprehensive information pertaining to Models 1a, 1b, and 1c.

- **Abstract:** Line 30 define past years. How authors concluded this because last 12 months was used in the data analyses. Conclusion needs improvements.

**Response:**

Thank you for noticing this inconsistency. We used “past 12 months” throughout the document.

- **General:** The manuscript uses the terms domestic violence and sexual violence interchangeably, which creates conceptual confusion. Since the findings specifically pertain to sexual violence, the terminology should be standardized throughout the text to avoid misinterpretation. Furthermore, the title should be revised to explicitly reflect that the study focuses on sexual violence, ensuring consistency between the reported results and the manuscript’s framing.

**Response:**

Thank you for this comment. We elected to use the term *intimate partner violence (IPV)* exclusively rather than *domestic violence*. IPV encompasses three forms of violence: sexual IPV, physical IPV, and emotional IPV. We applied this terminology consistently throughout the document, including the title.

Implementing these improvements will help ensure the manuscript is more polished, precise, and aligned with scholarly standards.

women’s polygyny status
