# Supplementary material for: Intimate partner violence and unmet need for family planning in the Democratic Republic of the Congo: A secondary analysis of the moderating role of reproductive coercion using performance monitoring for action data
Source: PLoS One. 2026 May 5;21(5):e0331236. doi: 10.1371/journal.pone.0331236 (PMC13143080; doi:10.1371/journal.pone.0331236)
Supplement: Attachment — Submitted filename: Responses to reviewers3.docx [file pone.0331236.s009.docx]

**RESPONSE TO THE ACADEMIC EDITOR:**

1. The **title** of the manuscript should be rewritten to include the study design

**Response:** The new proposed title is:

"Intimate Partner Violence and Unmet Need for Family Planning in the DRC: A Secondary Analysis of the Moderating Role of Reproductive Coercion using PMA Data"

We believe this version more accurately describes the methodology while maintaining the focus on our key findings.

We hope this revised title meets your expectations and remain at your disposal for any further information and suggestion.

women’s polygyny status
