# Supplementary material for: Intimate partner violence and unmet need for family planning in the Democratic Republic of the Congo: A secondary analysis of the moderating role of reproductive coercion using performance monitoring for action data
Source: PLoS One. 2026 May 5;21(5):e0331236. doi: 10.1371/journal.pone.0331236 (PMC13143080; doi:10.1371/journal.pone.0331236)
Supplement: Attachment — Submitted filename: Responses to reviewers4.docx [file pone.0331236.s010.docx]

**RESPONSE TO THE ACADEMIC EDITOR:**

1. The **title** should not contain any abbreviations.

**Response:** As requested, we have removed the abbreviation from the title. The revised title is:

"Intimate Partner Violence and Unmet Need for Family Planning in the Democratic Republic of the Congo: A Secondary Analysis of the Moderating Role of Reproductive Coercion Using Performance Monitoring for Action Data"

We hope this revised title meets your expectations and remain at your disposal for any further information and suggestion.

women’s polygyny status
